# Hybrid analysis of gene dynamics predicts context-specific expression and offers regulatory insights

**DOI:** 10.1093/bioinformatics/btz256

**Published:** 2019-04-17

**Authors:** Justin D Finkle, Neda Bagheri

**Affiliations:** 1 Interdisciplinary Biological Sciences, Northwestern University, Evanston, IL 60208, USA; 2 Department of Chemical and Biological Engineering, Northwestern University, Evanston, IL 60208, USA; 3 Center for Synthetic Biology, Northwestern University, Evanston, IL 60208, USA; 4 Chemistry of Life Processes, Northwestern University, Evanston, IL 60208, USA

## Abstract

**Motivation:**

To understand the regulatory pathways underlying diseases, studies often investigate the differential gene expression between genetically or chemically differing cell populations. Differential expression analysis identifies global changes in transcription and enables the inference of functional roles of applied perturbations. This approach has transformed the discovery of genetic drivers of disease and possible therapies. However, differential expression analysis does not provide quantitative predictions of gene expression in untested conditions. We present a hybrid approach, termed Differential Expression in Python (DiffExPy), that uniquely combines discrete, differential expression analysis with *in silico* differential equation simulations to yield accurate, quantitative predictions of gene expression from time-series data.

**Results:**

To demonstrate the distinct insight provided by DiffExpy, we applied it to published, *in vitro*, time-series RNA-seq data from several genetic *PI3K/PTEN* variants of MCF10a cells stimulated with epidermal growth factor. DiffExPy proposed ensembles of several minimal differential equation systems for each differentially expressed gene. These systems provide quantitative models of expression for several previously uncharacterized genes and uncover new regulation by the *PI3K/PTEN* pathways. We validated model predictions on expression data from conditions that were not used for model training. Our discrete, differential expression analysis also identified *SUZ12* and *FOXA1* as possible regulators of specific groups of genes that exhibit late changes in expression. Our work reveals how DiffExPy generates quantitatively predictive models with testable, biological hypotheses from time-series expression data.

**Availability and implementation:**

DiffExPy is available on GitHub (https://github.com/bagherilab/diffexpy).

**Supplementary information:**

Supplementary data are available at *Bioinformatics* online.

## 1 Introduction

Aberrant regulation of gene expression is frequently associated with diseases; thus, changes to gene expression serve as key proxies to infer cell state (Emilsson *et al.*, 2008). Differential gene expression analysis quantifies changes in gene expression between cell states. Expression is compared between genetically different cells, cells exposed to different exogenous treatments—such as small molecules, proteins, temperatures or other environmental cues—or a combination of several treatments. Each gene in the analysis is then categorized as a differentially expressed gene (DEG) or not. This categorization is often based on the magnitude of the log-fold change (LFC) of its expression between experimental conditions and by an adjusted *P*-value. DEGs are often split into groups of genes that are overexpressed or underexpressed (Law *et al.*, 2014; Pimentel *et al.*, 2017). Finding enriched Gene Ontology (GO) terms or pathways associated with the DEGs can elucidate the functional role of the experimental condition (Consortium, 2016; Subramanian *et al.*, 2005).

Measuring and analyzing the dynamics of gene expression are also critical to understanding responses involved in DNA repair, development and circadian rhythms (Hafner *et al.*, 2017; Jiang *et al.*, 2000, 2015). A typical time-series, gene expression experiment compares expression between experimental conditions over several time points (Hafner *et al.*, 2017; Schulz *et al.*, 2012). Many algorithms that identify DEGs from time-series data exist, but these algorithms focus on DEG identification for subsequent enrichment analyses (Spies *et al.*, 2017). Other algorithms use time-series expression data to infer the structure of gene regulatory networks (Finkle *et al.*, 2018; Madar *et al.*, 2009; Zoppoli *et al.*, 2010), or attempt to identify transcription factors (TFs) that explain changes in time-series gene expression, by pairing the expression data with ChIP-seq data (Mina *et al.*, 2015; Schulz *et al.*, 2012).

A limitation of existing differential expression analyses—both of static and time-series data—is that they do not propose quantitative models of a gene’s expression that can be tested in new experimental conditions. For example, if a gene is overexpressed in cells treated with a particular drug (compared with untreated cells), existing analyses cannot predict if that gene will be overexpressed, underexpressed or unchanged when a different drug is applied. Researchers can only infer how the regulation might occur and qualitatively predict how expression will differ in untested contexts.

Distinct from statistical enrichment approaches, differential equation models aim to use mechanistic information to describe how species, such as genes or proteins, interact and are well-suited to quantitatively predict gene expression in untrained conditions. However, designing and fitting differential equation parameters requires sufficient data; therefore, such models only exist for a few well-studied systems (Gambin *et al.*, 2013; Orton *et al.*, 2005; Pappalardo *et al.*, 2016; Sulaimanov *et al.*, 2017). Genetic and sparse regression algorithms can generate differential equation models directly from data, but current gene expression technologies cannot produce the highly sampled, low-noise data that these algorithms require (François and Hakim, 2004; Mangan *et al.*, 2016).

Data-driven methods to generate models that can quantitatively predict gene expression are currently limited. Network inference methods generate genome-scale models, however, to predict the expression of any one gene requires knowing the expression of several others (Bansal *et al.*, 2006; Finkle *et al.*, 2018; Geurts *et al.*, 2018). Other methods explicitly fit expression to a time variable, which ignores the molecular contexts driving expression (Hafner *et al.*, 2017). To fill this gap, we present Differential Expression in Python (DiffExPy), a framework that uses time-series expression data to create dynamical-systems models of gene expression.

**Fig. 1.**
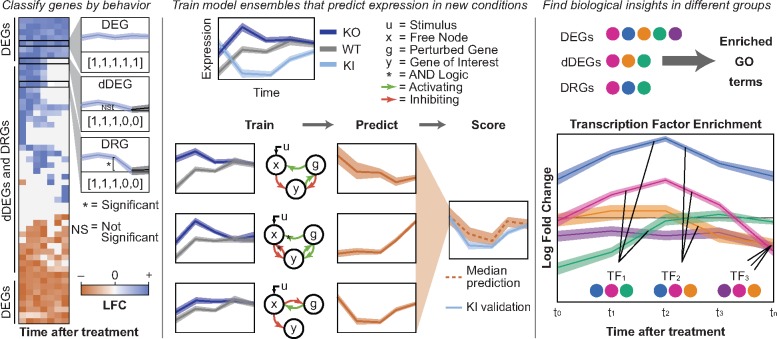
Overview of DiffExPy analysis. (Left) Genes are categorized as differentially expressed genes (DEGs), dynamic DEGs (dDEGs) or differentially responding genes (DRGs) from time-course gene expression data. Discrete responses (in brackets) are determined for each contrast. (Center) Stochastic differential equation (SDE) systems that match the dDEG gene profiles are selected from a library of possible models and combined into an ensemble model. The ensembles can predict gene expression behavior in new, untested conditions. (Right) Biological insights are gained by associating GO terms with gene classifications and associating TFs with groups of genes that share discrete differential behavior

DiffExPy first determines a discrete response from the expression of each gene in the time series based on the sign and significance of the gene’s LFC between conditions at each time point. Next, DiffExPy simulates time-series expression data from a library of minimal stochastic differential equation (SDE) systems that mimic the experimental conditions. Then, the discrete response of a gene is matched to models in the simulation library to train an ensemble model. This trained model can predict that gene’s expression in new conditions. DiffExPy also clusters genes by discrete response, and infers the timing of regulatory events by associating these gene groups with TFs and GO terms (Fig. 1).

We demonstrate the efficacy of DiffExPy on publicly available RNA-seq data from the GeneExpressionOmnibus(GEO) repository, accession number GSE69822 (Kiselev *et al.*, 2015). Previous analysis of this dataset further elucidated the transcriptional roles of phosphoinositide 3-kinase (*PI3K*) and phosphatase and tensin homolog (*PTEN*), which respectively phosphorylate and dephosphorylate phosphatidylinositidol-4, 5-bisphosphate (PIP2) to and from phosphatidylinositidol-3,4,5-trisphosphate (PIP3). PIP3 regulates many downstream pathways, most notably the *AKT* pathway (Kiselev *et al.*, 2015). For our analyses, we use data from the wild-type (WT), *PTEN* knockout (*PTEN* KO), A66-treated cells and *PI3K* knockin (PIK3CA H1047R) conditions. A66 inhibits the p110*α PIK3CA* and we refer to it as the inhibited condition (*PI3K*^inh^). The histidine-to-arginine substitution makes PIK3CA constitutively active, and we refer to it as the knockin (*PI3K* KI) condition. In the original study, expression was measured from MCF10a cells, a commonly used human breast epithelial cell line, stimulated with epidermal growth factor (EGF) using RNA-seq in three replicates at 0, 15, 40, 90, 180 and 300 min after EGF stimulation (Kiselev *et al.*, 2015).

Using the differential-expression data between the *PI3K*^inh^ and WT conditions, we train ensemble models for several genes. We validate the expression predictions for each gene using the *PI3K* KI time-series data and provide a straightforward approach to rank the confidence of each trained ensemble. The ensembles vary in size and consist of minimal SDE systems with different connectivities. We highlight results of three genes known to interact with the *PI3K* pathway that currently lack quantitative models for their expression. In doing so, we demonstrate how the trained ensemble models provide simple starting models for less-studied genes. We also use the discrete response calculated by DiffExPy to identify the timing of regulation by suppressor of zeste 12 (*SUZ12*) and forkhead box A1 (*FOXA1*) on their target genes.

DiffExPy is distinct from the status quo in generating dynamical system models *de novo* for many genes that were not previously characterized. Currently, DiffExPy is limited in that it constructs small models based on time-series data from individual perturbations. However, DiffExPy is readily extensible and can be adapted to other differential-expression packages, model assumptions and genomic data. For example, future improvements to DiffExPy could be made to incorporate multiple perturbations, additional omics data types and prior knowledge. Our work provides a foundation on which more complex models of gene expression can be developed.

## 2 Materials and methods

Using time-series RNA-seq data, DiffExPy sorts genes according to their discrete, dynamic, differential gene expression profiles (Fig. 1 and Supplementary Fig. S1). Each gene’s discrete profile is used to train an ensemble model of minimal SDE systems that predict expression in new conditions (Fig. 1). DiffExPy also associates GO terms with resulting groups, which suggest functional roles for the genes in each distinct cluster. Finally, DiffExPy associates TFs with genes that exhibit similar responses at specific times (Fig. 1). Overall, DiffExPy identifies (i) minimal dynamical systems models that accurately predict gene expression dynamics in untrained conditions, (ii) specific GO terms associated with classes of expression dynamics and (iii) specific TFs associated with genes with similar expression responses.

### 2.1 DiffExPy assigns discrete differential responses

To match gene expression responses to dynamical systems models, DiffExPy first calculates discrete responses from LFC contrasts generated by differential expression analysis (Supplementary Fig. S1) using the package *limma* (Ritchie *et al.*, 2015). A contrast is defined as a comparison of expression between conditions, time points or both. The discrete response is derived from the LFC value for a contrast, which can be positive (+1), negative (−1) or not significant (0). We assign gene labels based on discretized LFC values (Fig. 1 and Supplementary Fig. S1) as DEGs, dynamic DEGs (dDEGs) and differentially responding genes (DRGs).

DEGs are genes that are differentially expressed between conditions at one or more time points after the treatment, based on an *F*-test. dDEGs define the subset of DEGs that exhibit dynamic, or variable, differential expression across time. For instance, an expression profile need not be differentially expressed at time 0, but it can become differentially expressed at a later time point. DRGs contain at least one time point in which the LFC is significantly different from the LFC either at the previous time point (i.e. ${\text{LFC}}_{t}\neq {\text{LFC}}_{t-1}$) or from the time the treatment is applied (i.e. ${\text{LFC}}_{t}\neq {\text{LFC}}_{0}$), where significance is determined using an *F*-test. Classification of a gene as a dDEG or DRG is not mutually exclusive, and by definition, a dDEG or DRG is also a DEG.

#### 2.1.1 Definition of gene expression contrasts

A gene expression contrast compares the distribution of expression values of a gene between samples (Ritchie *et al.*, 2015). Using time-series data, the basic set of values used in a contrast for gene *i*, given condition *c*, with *R* replicates and at time *t* is defined as:

(1) $${\vec{g}}_{i|c}^{t}=[g_{i}^{1,t},g_{i}^{2,t}\ldots ,g_{i}^{R,t}]$$

The LFC is calculated as the ratio of the mean log_2_ expression value between the conditions:

(2) $$l_{i}^{t}=\frac{\langle {\vec{g}}_{i|\;\text{exp}\;}^{t}\rangle }{\langle {\vec{g}}_{i|\text{ctrl}}^{t}\rangle }$$

where *exp* is the experimental condition and *ctrl* is the control condition. By convention, the control condition is in the denominator, so positive LFC values correspond to overexpression in the experimental condition. For each contrast, a corresponding *P*-value is calculated. When multiple contrasts are made, an overall significance level is also calculated using an *F*-test. Significance levels are corrected for multiple hypothesis testing (Ritchie *et al.*, 2015). Differential expression calculations between genes are linearly independent and are easily extended to matrix form.

DiffExPy departs from the status quo by using time-series data to create more complex contrasts. Pairwise (PW) contrasts compare expression between experimental conditions at each time points. Time-series (TS) contrasts compare expression between a time point and the previous time point. Autoregressive (AR) contrasts compare expression between a time point and the time point before the treatment was applied. A detailed description of these and other combinations of contrasts (PW-TS and PW-AR) is available in Supplementary Figure S1.

#### 2.1.2 Discrete expression responses

To facilitate downstream analyses, DiffExPy calculates a discrete response for each gene based on the *P*-values and signs of LFC for the individual contrasts. If the *P*-value for a contrast is above the user-specified threshold, the LFC is not considered significant and is set to zero. The discrete response for gene *i* is defined as ${\vec{d}}_{i,x}=[d(l)]\;\forall \;l\in {\vec{l}}_{i,x}$, where *x* is one of the set {PW, TS, AR, PW-TS or PW-AR}. The discrete values are calculated using the signs of the LFC values as follows:

(3) $$d(l)=\{\begin{array}{cc} 1 & \text{sign}(l)>0\;\text{and}\;p(l)<p_{\text{cut}}\\ 0 & p(l)\geq p_{\mathrm{cut}}\\ -1 & \text{sign}(l)<0\;\text{and}\;p(l)<p_{\text{cut}} \end{array},$$

where *p*(*l*) is the adjusted *P*-value of the LFC for the contrast and *p*_cut_ is the significance threshold. For a time series of *T* time points, each discrete response has $3^{T-1}$ possible clusters—except for ${\vec{d}}_{\text{PW}}$, which has 3^*T*^. We did not filter LFC values by magnitude, but the option is provided in DiffExPy. We used a *P*-value threshold of 0.05 for all of our tests. A lower *P*-value cut-off could result in discrete responses with more zero values.

### 2.2 Training predictive models of gene expression

DiffExPy uses GeneNetWeaver (GNW) to generate minimal differential equation models for unique, three-node networks and to carry out stochastic simulations. GNW models include a protein and mRNA component (Schaffter *et al.*, 2011). Each model is an abstract representation of the flow of information that might regulate a gene’s expression and should not be used to identify specific regulation between genes. To mimic the experimental data, we used DiffExPy to conduct three independent, stochastic runs of each model and sampled each model at the same time points as the RNA-seq data. Microarray-like measurement noise was added to the data, and values were normalized between 0 and 1. A complete description of model generation is available in Supplementary Material.

Simulations were conducted under three genetic conditions: WT, knockout and knockin. It is important to note that the identity of the perturbed gene does not need to be known to gain information from DiffExPy. If a treatment with an unknown target is applied, DiffExPy can still provide insight into possible motifs of which a gene is a part that results in the observed expression behavior.

#### 2.2.1 Matching models to genes

After simulation, each SDE model had time-series data mimicking the experimental data. We conducted the same discrete clustering process on the simulated data. Each gene was matched to all SDE models with the same discrete response as the gene ${\text{match}}_{i}=\{m\in M|l_{i}^{t}=l_{m}^{t}\;\forall \;t\in T\}$, where *M* is the set of all models in the simulation library and *T* is the number of time points in the discrete response.

### 2.3 Model predictions

Each SDE model matched by DiffExPy can then simulate time-series data under different conditions to generate predicted LFC values at each time point, represented as a *T*-length vector ${\hat{l}}_{k}=[{\hat{l}}^{1},\ldots ,{\hat{l}}^{T}]$, where *k* is the index of the matched SDE system and *T* is the number of time points in the predicted time series. The time series of the control condition provides an internal control for predicting the response.

For our predictions, we applied a simulated knockin of *PI3K* to each of the trained SDE models as this matches the *PI3K* KI perturbation that was applied in the experimental data. Importantly, predictions to match different treatment strategies can also be made—such as targeting multiple nodes in the SDE models, inhibiting interactions between SDE nodes or changing the forcing function.

We found no feature of the individual models that correlated with their prediction error. We therefore created an ensemble prediction by using the median predicted LFC. For each gene *i*, the predicted LFC is calculated as the median LFC of all matched SDE predictions for each time *t*:

(4) $${\hat{l}}_{i}^{t}=\text{median}([{\hat{l}}_{m}^{t}\;\forall \;m\in {\text{match}}_{i}]).$$

The simulated model predictions in log_2_ expression space are ${\hat{y}}_{i}^{t}=b_{i}^{t}+{\hat{l}}_{i}^{t}$, where $b_{i}^{t}$ is the log_2_ expression of the control condition at time *t*, ${\hat{l}}_{i}^{t}$ is the model predicted LFC and ${\hat{y}}_{i}^{t}$ is the predicted log_2_ expression of the experimental condition.

#### 2.3.1 Scoring prediction accuracy

We validated the accuracy of the quantitative predictions by calculating the error between *PI3K* KI expression of a gene and its corresponding model’s prediction. We define accuracy as the mean-squared error (MSE) between a model’s average LFC (of three stochastic runs) and the true LFC. MSE values range from 0 to *∞*, where smaller values indicate that the prediction is closer to the true LFC value. We know of no existing, data-driven method that generates models capable of quantitative, time-series, gene expression prediction to provide an appropriate basis for comparison. Thus, we used the selection of a random model from our library as the null model comparison.

## 3 Results

### 3.1 Many previously uncharacterized genes are matched to ensemble models

We used the discrete response profiles to match each gene to an ensemble of three-node SDE models that each share similar dynamics upon simulation. Our library consists of 2172 uniquely structured SDE models. We trained the models using the *PI3K*^inh^ and WT data, and we used the *PI3K* KI data as test data to validate the predictions. Simulations for each network model were created to match the *PI3K* genetic condition and EGF stimulation. The simulated data are sampled at the same time points used in the experiments. Details of the library creation are provided in Supplementary Material.

As the *PI3K*^inh^ does not affect the expression of many genes (Kiselev *et al.*, 2015), DiffExPy only identifies 223 dDEGs from the differential expression analysis of the *PI3K*^inh^ and WT data. There is a many-to-many match between the dDEGs and the possible three-node SDE systems. A total of 217 of the dDEGs were matched to at least one network model. Of the 217 matched genes, few are well-studied; there is sparse information about their functional role. We identified just nine genes whose paralogs were likely to exist in current computational models of well-studied signaling pathways. These include genes in the *MAPK*, *JAK/STAT* and *PI3K/AKT/mTOR* pathways (Gambin *et al.*, 2013; Orton *et al.*, 2005; Pappalardo *et al.*, 2016; Sulaimanov *et al.*, 2017).

### 3.2 Ensemble models highlight possible dynamical systems from which to build more detailed models

**Fig. 2.**
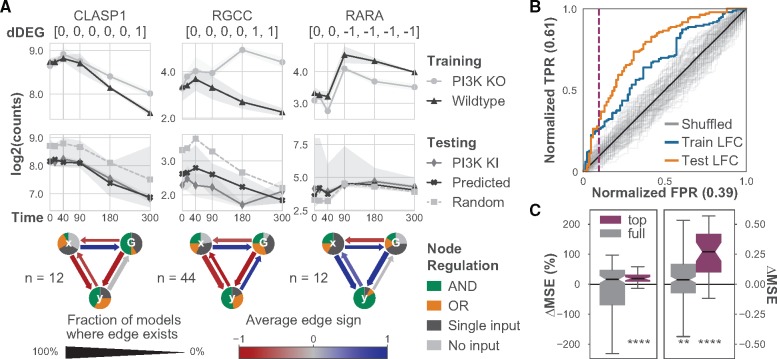
Ensembles of minimal SDE systems trained on *PI3K*^inh^ and WT data accurately predict expression in untrained *PI3K* KI condition. (**A**) Three examples of dDEGs with matching dynamical models. (A, top row) Normalized gene expression for each gene in *PI3K*^inh^ and WT used to train the models. The discrete response of the pairwise contrasts (in brackets) for each dDEG show when differential expression occurs. (A, middle row) Normalized gene expression for model predictions, null model predictions and true *PI3K* KI expression. Trained and null model predictions are median values, and the filled regions show the 83% CI of the median. (A, bottom row) Network diagrams summarize the ensemble models matched to each gene in the training condition. (**B**) AUROC curve plot of different methods for sorting gene predictions. Sorting by mean LFC between the training conditions places more accurate predictions at the top of the list. A threshold for selecting more accurate predictions (purple, dashed line) is calculated using the elbow rule of the sorted mean LFC values in the training condition (Supplementary Fig. S7). (**C**) Box plots show the normalized and absolute difference in MSE of the trained models compared with the paired random models for all genes. The top set of genes (purple) were determined by the elbow rule and are significantly more likely to generate more accurate predictions. *P*-values were calculated using the Wilcoxon signed-rank test. **P < 0.01, ****P < 0.0001

Each independent model in the ensemble suggests a possible SDE system whose simulations match the qualitative features of the experimentally measured expression. Specifically, each SDE system represents how the gene of interest (node *y*) might interact with the perturbed gene (node *G*) and the rest of the genome (node *x*). In this experiment, *G* represents *PI3K* as it was the knocked-out, knocked-in or inhibited gene. Summaries of the models that match each gene and create the quantitative predictions reveal possible regulatory interactions that result in the observed dynamics (Fig. 2A). We highlight results of trained models for three genes that exhibit different discrete responses and predictive accuracy: cytoplasmic linker-associated protein 1 (*CLASP1*), regulator of cell cycle (*RGCC*) and retinoic acid receptor alpha (*RARA*). *CLASP1* and *RARA* were previously shown to interact with components of the *PI3K/AKT* pathway (Lansbergen *et al.*, 2006; Srinivas *et al.*, 2006).

Models for *CLASP1* and *RGCC* primarily contain inhibition by both *x* and *G*, whereas *x* and *G* appear as activators of *RARA*. Furthermore, in almost all models matched to *RARA*, both *x* and *G* must be present to activate *RARA*. The *RGCC* models often contain activation of *G* by *RGCC*. Conversely, models of *CLASP1* and *RARA* often exhibit feedback on *G* but are not consistently activating or inhibiting. Overall, these models suggest modes of regulation between *PI3K* and *CLASP1, RGCC* and *RARA*, and predict gene responses to future conditions and treatments. These models also provide a basis from which more detailed models can be developed.

### 3.3 DiffExPy ranking sorts models by predictive accuracy

Because every ensemble model is not equally predictive of its respective gene, we searched for a metric to rank model predictions. We find that the mean absolute LFC between the *PI3K* KI and WT conditions correlates with improved model accuracy (Spearman’s *ρ  *=  0.636, *P *=* *5.4e-26, Supplementary Fig. S2). Ordering gene predictions by the mean absolute LFC places genes with lower MSE at the top of the list. Treating genes with positive ΔMSE (i.e. lower error than random) as positive classifications, we can assess the area under the receiver operator characteristic (AUROC) and area under precision recall (AUPR) curves. The AUROC for this ranking is 0.76 and the AUPR is 0.78, both of which are significantly greater than expected from a random ordering (Fig. 2B and Supplementary Fig. S3).

Since future experiments will not always include validation data, we sought a proxy for model confidence. We find that the mean absolute LFC between the *PI3K*^inh^ and WT correlates with the mean absolute LFC between the *PI3K* KI and WT (Spearman’s *ρ  *=  0.684, *P *=* *2.7e-31, Supplementary Fig. S4). The mean absolute LFC between the *PI3K*^inh^ and WT also correlates with improved model accuracy (Spearman’s *ρ  *=  0.418, *P *=* *1.4e-10, Supplementary Fig. S5). This ranking yields an AUROC of 0.66 and an AUPR of 0.73, which are slightly lower, but still significantly better than random (Fig. 2B and Supplementary Fig. S3).

We believe this sorting is intuitive. A gene with a greater effect size in the transcriptional response provides more information during training, which results in better matched models. On average, a random model predicts no LFC between WT and another condition for a given gene, so a trained model prediction should be more accurate (Supplementary Fig. S6).

### 3.4 Top-ranked genes offer accurate predictions

Overall, the predictions for the dDEGs have a median MSE that is 0.027 (*P *=* *0.018) lower than random (ΔMSE). However, after ranking genes by mean *PI3K*^inh^-WT LFC, we applied an elbow rule to select the top 40 genes. Our results indicate that the top-ranked genes have significantly more accurate predictions than random models. The top genes have a median ΔMSE of 0.523 (*P *=* *1.45e-6) and a %MSE of 33.3% (*P *=* *1.74e-5, Fig. 2C). The elbow rule gives an empirical threshold to select the top predictive genes (Supplementary Fig. S7).

### 3.5 Gene classifications from discrete responses associate with specific GO terms

**Fig. 3.**
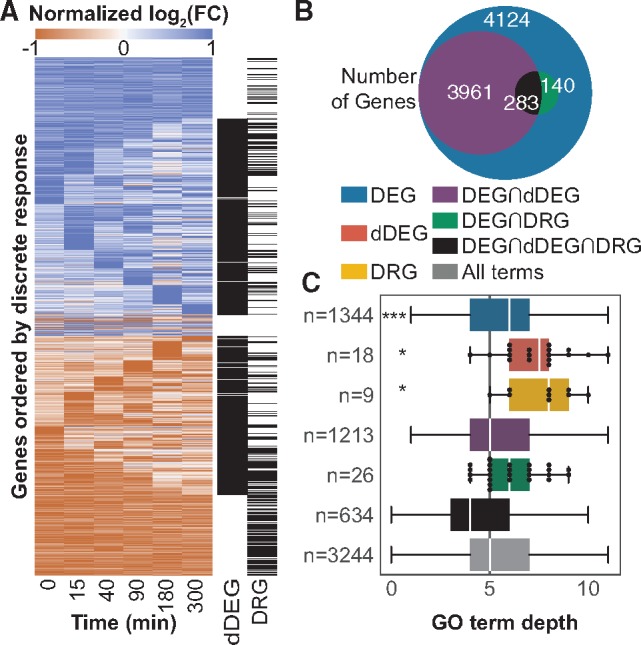
Summary of gene classifications comparing *PTEN* KO to WT. (**A**) Heatmap of row normalized LFC for all DEGs. Genes that are classified as dDEG or DRG are also labeled. (**B**) Overlap of genes that are classified as DEG, dDEG, DRG or some combination. By definition, all dDEGs and DRGs must also be DEGs. (**C**) Comparison of distributions of GO term depths uniquely associated with intersections of gene sets. Even though no genes are classified as only dDEG or DRG, these gene sets associate with unique, specific GO terms not found in the dDEG set. The number of unique terms associated with each set, *n*, is shown. *P*-values were calculated using a discrete KS test. *P < 0.05, ***P < 0.001

To demonstrate the high-level biological insights gained from the discrete responses, we present results of classifying genes from their discrete responses comparing the *PTEN* KO to WT time-series expression data. We identify 8508 DEGs, of which 3961 are classified as dDEGs, 140 as DRGs and 283 as all three (Fig. 3B). We performed GO term enrichment analysis on each of these non-mutually exclusive gene classifications. Enriched GO terms were grouped by the exclusive set to which they belonged. Thus, genes can have multiple labels, but GO terms can only be associated with one group. For example, a GO term associated with both the sets of DEGs and dDEGs—which have many overlapping genes—would be assigned to the DEGs∩dDEGs group, whereas a GO term only associated with the set of dDEGs would be assigned to the dDEG group (Fig. 3B and C).

All terms were called from the same directed acyclic graph (DAG). Term depth quantifies the level in the GO hierarchy, and it is used as proxy for term specificity. Even though no genes are categorized exclusively as dDEGs or DRGs, there are very specific terms associated only with these groups (Fig. 3C). Results of the gene classifications for the *PI3K* KI and *PI3K*^inh^ compared with WT are provided in Supplementary Figures S8 and S9. Specific gene clusters with similar discrete responses may also be used for GO enrichment analysis, but we next focus on using them for TF enrichment.

### 3.6 TF enrichment suggests regulators of gene expression

Similar to GO term enrichment analysis, we calculate TF enrichment for gene clusters. A group of genes enriched for association with a particular TF may indicate that the TF is responsible for the observed change in expression. Existing methods, such as weighted gene co-expression network analysis (WGCNA) and dynamic regulatory events miner (DREM), perform clustering of gene profiles for subsequent GO and TF enrichment analysis (Langfelder and Horvath, 2008; Schulz *et al.*, 2012; Wise and Bar-Joseph, 2015). In contrast, DiffExPy uses the discrete LFC values (0, 1, −1) to generate default clusters. This discretization enables grouping genes in various ways that suggest different types of coregulation by a shared TF.

For example, the set of all DRGs is enriched for association with 52 TFs (adjusted *P *<* *0.05). This set includes supressor of zeste 12 (*SUZ12*) and forkhead box A1 (*FOXA1*), which were not identified in the original study (Kiselev *et al.*, 2015). *SUZ12* is a zinc finger protein and a component of the polycomb repressive complex 2 (PRC2). PRC2 has histone methylation activity, yet its regulatory role in cell fate is uncertain (Comet *et al.*, 2016; Squazzo *et al.*, 2006). *FOXA1* is an important TF in breast and prostate cancers and is known to be a target of both MAPK and AKT (Bernardo *et al.*, 2013; Potter *et al.*, 2012).

**Fig. 4.**
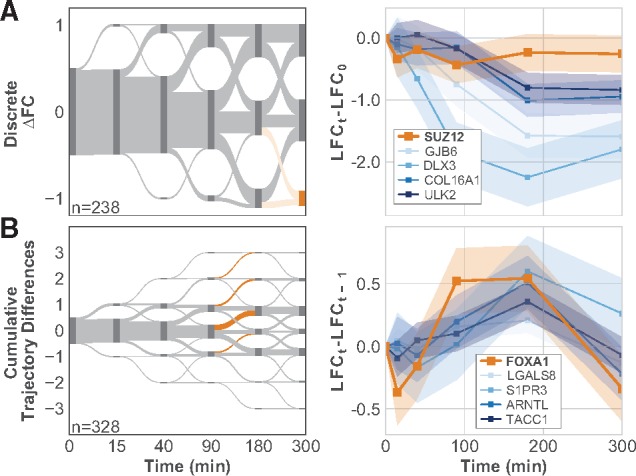
Specific timing of changes in gene expression identifies possible regulators. (**A**, left) Sankey plot of discrete FC between *PTEN* KO and WT compared with FC before the stimulus is applied shows when DRGs respond to the stimulus. Nodes (dark gray) are scaled by the number of genes in that state. Edges (light gray) show the fraction of genes moving from one state to another between time points. The node and edges highlighted in orange show the set of genes associated with *SUZ12*. (A, right). LFC values relative to LFC before stimulus for *SUZ12* and several genes associated with *SUZ12*. (**B**, left) Sankey plot of the cumulative differences in *PTEN* KO slope and WT slopes. Segments highlighted in orange show genes whose KO expression increases more than their WT expression between either 90 and 180 min or 180 and 300 min. These genes are enriched for association with *FOXA1*. (B, right) LFC values relative to LFC at the previous time point for *FOXA1* and several associated genes. Shaded regions in time-series plots show the 83% confidence intervals

Using the temporal information inherent to a time-series dataset, we can identify when, and how, the regulation by these factors occurs. For example, we identify the set of 41 genes that have lower LFC at 300 min than at 0 min, which are enriched for association with *SUZ12* (Fig. 4A). A total of 13 of the 41 genes are known to be associated with *SUZ12*, and the enrichment suggests that changes in expression for this group are regulated by *SUZ12*. Interestingly, there is no identifiable change in *SUZ12* expression (Fig. 4A), indicating that downstream gene regulation by *SUZ12* might depend on post-trancriptional changes, such as sumoylation (Riising *et al.*, 2008).

We also find *FOXA1* to be associated with genes that show an increase in LFC between 90 and 180 min. A natural hypothesis is that this group of 79 genes all show the same change in expression at these later time points because they share a common regulator, *FOXA1* (Fig. 4B). In contrast to *SUZ12*, *FOXA1* exhibits a distinct differential response beginning 90 min after the EGF stimulus. Several of the genes that have the described behavior and are associated with *FOXA1* show a similar qualitative differential response to EGF as *FOXA1*. These results suggest that *FOXA1* might regulate the expression of these genes, as well as others in the set, in response to EGF stimulation. Additionally, each of these genes, including *FOXA1*, is classified as a DRG, further supporting the hypothesis that *PTEN* is required for proper expression of these genes in response to EGF stimulation.

*SUZ12* and *FOXA1* are not the only TFs associated with discrete response clusters. Instead, these examples demonstrate two possible ways the discrete analysis might identify enriched regulators for groups with different response behaviors. The timing of the expression behavior creates strong, testable hypotheses for the inferred regulators. Additional enrichment results, and a complete discussion of the enrichment methods, are provided in Supplementary Material.

## 4 Discussion

The characterization of less-studied genes is fundamental to understanding cellular responses in diverse environmental contexts (Stoeger *et al.*, 2018). In this study, we presented DiffExPy, an analytical framework that calculates discrete differential expression responses and trains dynamical systems models for many quantitatively uncharacterized genes.

We demonstrated how, for each matched gene, DiffExPy prototypes quantitative models that offer accurate predictions of gene expression in untrained conditions. We also validated the DiffExPy model predictions of each gene’s expression in the untrained *PI3K* KI condition (Fig. 2). Our results suggest that *PI3K* inhibits expression of both *CLASP1* and *RGCC*, often in conjunction with additional factors. These *de novo* results are supported by previous experiments. *CLASP1* was shown to interact with proteins affected by *PI3K* activity (Lansbergen *et al.*, 2006). *RGCC* was also demonstrated to have several regulatory roles in the *PI3K* pathway (Tegla *et al.*, 2015). Discrepancies with other data can refine the models and our understanding of each gene’s regulation. For example, the summary of *RARA* suggests that for the observed response, *PI3K* is a positive regulator of *RARA*. This result is surprising because *PI3K* activates AKT, which was shown to subsequently inhibit *RARA* (Srinivas *et al.*, 2006). One explanation is that an unknown activating path between *PI3K* and *RARA* exists.

We also demonstrated how DiffExPy associates groups of similar, discrete gene expression responses with TFs, such as *SUZ12* and *FOXA1* (Fig. 4). Though *SUZ12* expression does not differ between the *PI3K*^inh^ and WT conditions, several known and possibly new targets of *SUZ12* exhibit a differential response to EGF stimulus. These observations might suggest that regulation by *SUZ12* results from post-translational modification (Riising *et al.*, 2008). Conversely, *FOXA1* and many of its targets exhibit a differential response, which is consistent with previously studied interactions between *FOXA1* and the AKT pathway (Bernardo *et al.*, 2013; Potter *et al.*, 2012). Finally, we show how each gene classification associates with GO terms that enable a unique understanding of regulatory functions.

DiffExPy was formulated to create predictive models for many genes with varied dynamic expression responses. We limited our model library to three-node gene regulatory networks without self-edges (Supplementary Fig. S10). As such, a suitable model match might not exist in the library for each gene. In our analysis, a small fraction of genes (6 of the 223 dDEGs, or less than 3%) did not match to a suitable model. The absence of matches might be attributed to the limited scope of SDE models in the library, limiting possible gene expression dynamics. The library could be expanded to include four-node networks, which might be capable of simulating more qualitatively diverse expression dynamics. Expanding the library might be computationally expensive and require optimizing the library generation step, simulation and matching. Additionally, the current SDE models could be simulated with different kinetic assumptions, though the accuracy of these assumptions would need to be validated. Finally, because our training and testing perturbation affect the same gene, the ranking of the results might not hold for all predictions. We also focused only on comparisons between pairs of experimental conditions (i.e. only one node is perturbed). Integrating multiple experimental conditions into model training might yield more predictive models, but also make training the models more difficult.

A complete understanding of cellular regulation cannot be gained only from transcriptomics. Epigenomic data from ChIP-seq, ATAC-seq, etc., can provide additional information to increase the mechanistic specificity of the models by identifying direct regulators and chromatin accessibility. Currently, TF enrichment is calculated using associations derived from ENCODE (Schulz *et al.*, 2012). Direct measurements of TF association or chromatin accessibility, during the same time course, could be directly integrated into the existing framework. Unfortunately, this data was not available for our analysis and might currently be cost prohibitive.

Overall, few genes have detailed biochemical models that quantitatively predict their behavior in diverse conditions. Characterizing how less-studied genes are regulated in multiple contexts will improve our understanding and treatment of disease. The models generated by DiffExPy provide systematic, reliable starting points for quantitative models of regulation based on time-series, differential-expression data.

## Funding

This research was supported, in part, by a Nicholson Fellowship and Biotechnology Training Program Fellowship T32 GM008449 (to J.D.F.); NSF CAREER Award [CBET-1653315 to N.B.]; NIH NU-CCNE (U54 CA199091-03); and the McCormick School of Engineering.

*Conflict of Interest*: none declared.

## Supplementary Material

btz256_Supplementary_File
