# Supplementary material for: Hybrid analysis of gene dynamics predicts context-specific expression and offers regulatory insights
Source: Bioinformatics. 2019 Apr 17;35(22):4671–8. doi: 10.1093/bioinformatics/btz256 (PMC6853664; doi:10.1093/bioinformatics/btz256)
Supplement: btz256_Supplementary_File [file btz256_supplementary_file.zip › btz256-suppl_data/Supplementary-Information.pdf]

Supplementary Information

Hybrid analysis of gene dynamics predicts context specific expression and offers regulatory insights

Justin D. Finkle, Neda Bagheri

March 7, 2019

Supplementary Materials and Methods

Experimental data set

All code used in the analysis of gene expression, and all *in silico* data is available on GitHub (*https://github.com/bagherilab/diffexpy*). The *in vitro* data used in this study was previously published and is available in the Gene Of Expression (GEO) repository, with accession number GSE69822 [1].

Gene expression data representation

The measurement of expression for gene i with R replicates is defined by the R-length vector:

$$\vec{g_i} = [g_i^1, g_i^2 \dots, g_i^R]^\top \tag{1}$$

When gene expression is measured under different conditions for differential expression analysis, the measurement of expression for gene i, given condition c, with R replicates is expanded to the following R-length vector:

$$\vec{g}_{i|c} = [g_i^1, g_i^2 \dots, g_i^R]^\top \tag{2}$$

A gene expression experiment that measures N genes is represented as an $R \times N$ matrix:

$$\begin{aligned}\mathbf{E_c} &= [\vec{g}_{1|c}, \dots, \vec{g}_{N|c}] \\ &= \begin{bmatrix} g_1^1 & \cdots & g_N^1 \\ \vdots & \ddots & \vdots \\ g_1^R & \cdots & g_N^R \end{bmatrix}\end{aligned} \tag{3}$$

Time-series gene expression data representation

Time-series gene expression data is represented by vertically concatenating gene expression matrices for T time points into a $(T * R) \times N$ matrix as follows:

$$\begin{aligned}\mathbf{T_c} &= [\mathbf{E_c^1}, \dots, \mathbf{E_c^T}]^\top \\ &= \begin{bmatrix} g_1^{1,1} & \cdots & g_N^{1,1} \\ \vdots & \ddots & \vdots \\ g_1^{R,1} & \cdots & g_N^{R,1} \\ \vdots & \ddots & \vdots \\ g_1^{R,T} & \cdots & g_N^{R,T} \end{bmatrix}\end{aligned} \tag{4}$$

Here, each gene is sampled consistently. While the sampled time points do not need to be evenly spaced, each gene must be measured at the same time points.

Differential expression analysis

Computational tools and data normalization

The core differential expression analysis was conducted using the *rpy2* Python package to interface with the R packages *edgeR* and *limma* [2, 3]. Genes with low counts were removed using *DGElist* from *edgeR*. Counts were then prepared for differential expression analysis using *voom* [4]. *limma* expects log_2 transformed data, and data that does not conform to this expectation may yield unexpected results. DiffExPy is tailored to analyze time-series data, but the interface with R also enables users to conduct differential expression analysis with static data.

Definition of DiffExPy contrasts

DiffExPy makes contrasts along two dimensions, between conditions and between time points (SI Fig. S1). DiffExPy defines the following classes of contrasts:

1. **Pairwise (PW)** - calculate LFC at each time point between conditions:

$$\vec{l}_{i,PW} = [\log_2 \frac{\vec{g}^{\,1}_{i|exp}}{\vec{g}^{\,1}_{i|ctrl}}, \ldots, \log_2 \frac{\vec{g}^{\,T}_{i|exp}}{\vec{g}^{\,T}_{i|ctrl}}] \tag{5}$$

2. **Timeseries (TS)** - calculate LFC between each time point and the previous time point for one condition, c:

$$\vec{l}_{i,TS} = [\log_2 \frac{\vec{g}^{\,2}_{i|c}}{\vec{g}^{\,1}_{i|c}}, \ldots, \log_2 \frac{\vec{g}^{\,T}_{i|c}}{\vec{g}^{\,T-1}_{i|c}}] \tag{6}$$

3. **Autoregressive (AR)** - calculate LFC between each time point and the time point before the treatment is applied for one condition, c:

$$\vec{l}_{i,AR} = [\log_2 \frac{\vec{g}^{\,2}_{i|c}}{\vec{g}^{\,1}_{i|c}}, \ldots, \log_2 \frac{\vec{g}^{\,T}_{i|c}}{\vec{g}^{\,1}_{i|c}}] \tag{7}$$

   Here we assume the treatment is applied at time t=1. The first contrast is equivalent to that of the TS.

4. **Combinations** - The TS and AR contrasts can also be combined with the PW contrasts to assess if the LFC is significantly different between both the time points and the conditions.

   - **PW-TS** - calculate the LFC between conditions and the previous time point:

$$\vec{l}_{i,\text{PW-TS}} = [(\log_2 \frac{\vec{g}^{\,2}_{i|exp}}{\vec{g}^{\,2}_{i|ctrl}} - \log_2 \frac{\vec{g}^{\,1}_{i|exp}}{\vec{g}^{\,1}_{i|ctrl}}), \ldots, (\log_2 \frac{\vec{g}^{\,T}_{i|exp}}{\vec{g}^{\,T}_{i|ctrl}} - \log_2 \frac{\vec{g}^{\,T-1}_{i|exp}}{\vec{g}^{\,T-1}_{i|ctrl}})] \tag{8}$$

   - **PW-AR** - calculate the LFC between conditions and the time point before the treatment is applied:

$$\vec{l}_{i,\text{PW-AR}} = [(\log_2 \frac{\vec{g}^{\,2}_{i|exp}}{\vec{g}^{\,2}_{i|ctrl}} - \log_2 \frac{\vec{g}^{\,1}_{i|exp}}{\vec{g}^{\,1}_{i|ctrl}}), \ldots, (\log_2 \frac{\vec{g}^{\,T}_{i|exp}}{\vec{g}^{\,T}_{i|ctrl}} - \log_2 \frac{\vec{g}^{\,1}_{i|exp}}{\vec{g}^{\,1}_{i|ctrl}})] \tag{9}$$

Because the comparison is to the previous time point, for all comparisons except PW, there are T-1 contrasts. The combination contrasts, PW-TS and PW-AR, provide the most information about when a differential response to a treatment occurs between conditions. However, fewer significant differences are identified because the pooled variances decrease statistical power.

Gene classification

DiffExPy classifies each gene as a differentially expressed gene (DEG), dynamically differentially expressed gene (dDEG), or a differentially responding gene (DRG). Genes are considered DEGs if they pass an F-test described in the *limma* documentation [3]. We enforce that all genes classified as dDEGs and DRGs must also be DEGs. A gene is considered a dDEG if all of its contrasts do not have the same LFC sign, *i.e.* $|\{d \; \forall \; d \in \vec{d_x}\}| > 1$. This filter removes genes that are differentially expressed due to the genetic change but that are not affected by the stimulus. The classification of a DRG is more stringent. A gene is classified as a DRG if the adjusted p-value of the F-test was significant for one of $\vec{d}_{\text{PW-TS}}$ or $\vec{d}_{\text{PW-AR}}$.

There are many ways to assign genes to a cluster depending on the number of time points, treatments, and conditions over which gene expression is measured. Different methods of clustering test different hypotheses, and we leave flexibility for the user to decide what method is appropriate for their specific application.

Discrete response cluster scores

DiffExPy empirically ranks genes within a discrete cluster (Fig. S11). The cluster score for gene i is computed by calculating the fraction of the sum of the LFC values for each contrast that matches the discrete LFC sign values as follows:

$$CS_i = \frac{\sum\limits_{l \in \vec{l}_x} f(l,p,d)}{\sum\limits_{l \in \vec{l}_x} |l|}, \tag{10}$$

where $\vec{l}_x$ is the vector of LFC values for contrast class x, p is the p-value corresponding to each l, and d is the corresponding discrete value of the assigned cluster. The function f weights the calculated LFC by the apparent p value as follows:

$$f(l,p,d) = \begin{cases} |l(1-p)| - |l - l(1-p)| & sgn(l) = d \\ |l - l(1-p)| - |l(l-p)| & sgn(l) \neq d \end{cases} \tag{11}$$

Essentially, if a contrast is assigned a nonzero discrete value and the LFC value of the contrast is highly significant (low p-value), most of the LFC value is retained.

CS values are bounded between -1 and 1. Empirically, the cluster score sorts genes by how well they match the discrete path defined by the discrete cluster. It performs poorly for some edge cases, such as when all values in $\vec{d}$ are 0, but these were previously filtered out by the gene classification step.

Model library creation

We generated a library of minimal dynamical systems that was used to simulate time-series expression data that mimics experimental conditions.

Model connectivity

Each model is a directed network with three nodes. Node y represents the output that is matched to measured gene expression. Node G represents the gene that is perturbed, as with a knockout or knockin. Node x is an abstract node that serves two purposes. It summarizes the interactions between G and y with the rest of the genome, allowing for more complex regulatory motifs. Node x is also the target of the externally applied treatment (Fig. S10A). Edges between nodes either activate or inhibit the target node (Fig. S10B).

To limit the regulatory combinations, we prohibited self edges, which yielded 704 unique, weakly-connected networks. We added an input node u to each model to create a controlled forcing function, which represents the external treatment, on node x. The input u was linearly combined with regulation of x by the other nodes.

Model parameterization

We used GeneNetWeaver (GNW) to generate minimal differential equation models for each of the networks and carry out stochastic simulations. GNW models include a protein and mRNA component. Full details are available in the original paper [5]. Systems Biology Markup Language (SBML) style models were generated for each network structure with parameters assigned random values by GNW.

Model parameters were not optimized to improve the fit. It is unclear what objective function—such as minimizing error to LFC or matching expression distributions at each time—would be optimal to fit the parameters. While the parameter space is technically infinite, we relied on GNW to select biologically relevant parameters.

Multiple regulator logic

If a node has two coincident edges, the regulatory logic can be either AND or OR between them (Fig. S10D). However, GNW randomly assigns logic to the regulation of the target mRNA. For each of the 704 network structures, we generated SBML models for each combination of logic, resulting in a library of 2,172 network structure and logic combinations. This library represents a highly constrained structural search space. If the search space were increased to allow self edges, and all combinations of regulatory logic with two or three coincident edges were explored, there would be 102,356 unique models. If the search space included four node networks, an no self edges, there would be 4,870,752 unique networks. We did not simulate these large libraries.

Gene perturbation

For each base model, which we consider the WT model, we created corresponding KO and KI models (Fig. S10C). The modified models have the same parameters as the WT model with minor changes to the mRNA synthesis parameters for node G. In the KO model, the maximum transcription of G was set very low (1e-7), because a value of zero causes integration errors. In the KI model, all upstream regulation of G was removed, and the transcription rate was set to a constant value equal to its maximum rate in the WT model, representing constitutive expression.

Time-series simulations

Stochastic time-series simulations were carried out for each SBML file using GNW. Each stochastic differential equation system was sampled every minute for 1000 minutes. The stimulus was applied to node u at the start and removed halfway through the time series. DiffExPy then sampled the time series at intervals that matched the experimental data. Three independent, stochastic runs of each model were created to represent biological replicates. Microarray-like measurement noise was added to the data, and values were normalized between 0 and 1 [5].

Gene Ontology and transcription factor enrichment

An ontology is contructed as a directed acyclic graph (DAG), where the root node has the smallest term depth [6]. In general, more specific GO terms are further down the tree and therefore have higher term depths. Enriched GO terms were called for each class of genes—DEG, dDEG, and DRG—using the same GO DAG, which preserves the depth of the term relationships. We then calculated the term overlap between each combination of exclusive groups. For example the GO terms in the group $DEG \cap dDEG$ are associated with DEGs and dDEGs, but not associated with the DRGs. We calculated p-values to compare the distributions of term depths between groupings using a discrete KS test [7].

To calculate enrichment for associated TFs, gene lists were encoded as TFs using a dictionary of genes associated with each TF. We used a TF association dictionary derived from ENCODE data [8]. TF enrichment was calculated using Fisher's Exact test [9], implemented in *scipy*, comparing the TFs associated with a gene list to the TFs associated with a background gene list. We used different

lists of genes as the background depending on the hypothesis being tested. To identify enriched TFs in the set of DRGs, we used the set of all genes as the background. To identify the specific timing of TF enrichment of *SUZ12* and *FOXA1* we used the set of DRGs as the background.

The number of distinct discrete responses increases exponentially with the number of measured time points. For most current experiments, there are few time points and this will not be an issue. However, if more time points were available, specific qualitative behaviors could still be interogated. For example, one could ask "which transcription factors are associated with the set of genes that is initially not differentially expressed before treatment but becomes differentially expressed after X minutes?". The discrete clusters could easily be used to group genes with this behavior together. Genes A and B may have discrete responses *[0, 0, 0, 1, 1, 1]* and *[0, 0, 0, 0, 1, 1]*, but would be grouped together for enrichment analysis.

Computational development

DiffExPy was developed in Python 3.5.2 using the following major packages: *NumPy and SciPy* [10], *pandas* [11], and *rpy2*. Gene Ontology (GO) enrichment was calculated using the python library *goatools* [12]. The discrete KS test was conducted using the *dgof* package [7]. Figures were generated using *seaborn* and *matplotlib* [13]. The code for DiffExPy can be found on GitHub (*https://github.com/bagherilab/diffexpy*).

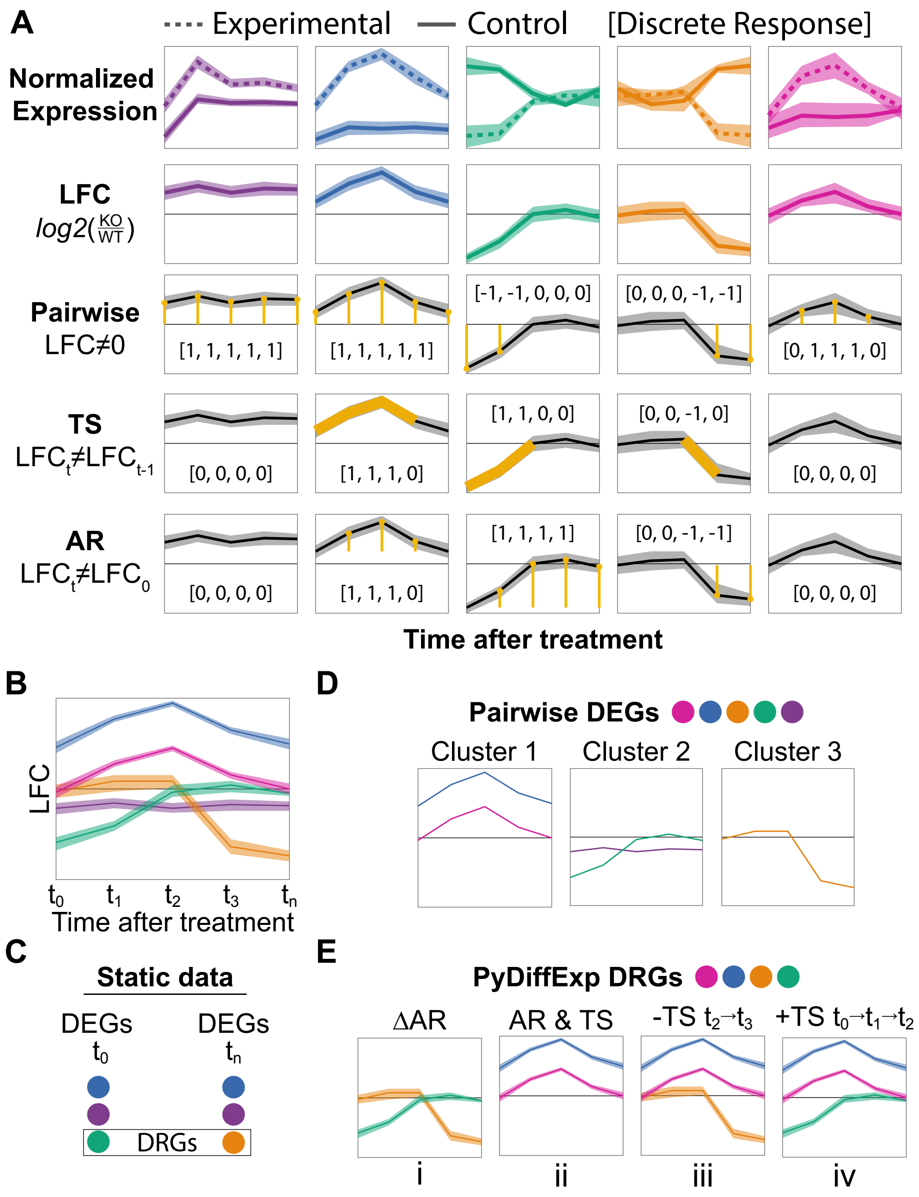

Fig. S1. Comparison of strategies to identify and cluster DEGs, dDEGs, and DRGs. (A) Overview of statistical contrasts. Normalized gene expression values, measured for each gene at several time points, were used to calculate the log fold change (LFC) between the experimental (dashed) and control (solid) condition. The lines show the mean expression or LFC, and the shaded area is the error (e.g. 95% confidence interval). Pairwise comparisons independently assess if LFC values are nonzero. Timeseries (TS) comparisons assess if the LFC changed significantly from the previous time point ($l_t \neq l_{t-1}$). Autoregressive (AR) comparisons check if the LFC is different than the initial LFC due to the experimental condition ($l_t \neq l_0$). The discrete responses corresponding to the type of contrast are displayed on the plots. (B) The LFC for example genes measured over time. (C) Sets of DEGs identified by comparing data only at individual time points. DRGs are only identifiable as those whose differential expression at t_0 is statistically different from t_n, which misses many genes. (D) Set of DEGs clustered into groups with similar mean LFC trajectories. DEGs are calculated using an F-test on all pairwise comparisons. Genes can subsequently be clustered by mean LFC using many available clustering methods, such as k-means. (E) Set of DRGs calculated by DiffExPy which includes genes with transient regulation (blue and magenta) that is not captured with only endpoint measurements. Genes that are consistently differentially expressed but show no differential response (e.g. purple) are not considered DRGs. DRGs can be clustered in many ways to find genes that have statistically different expression at later time points (i), have the same response trajectory (ii), or respond similarly at a specific time (iii and iv).

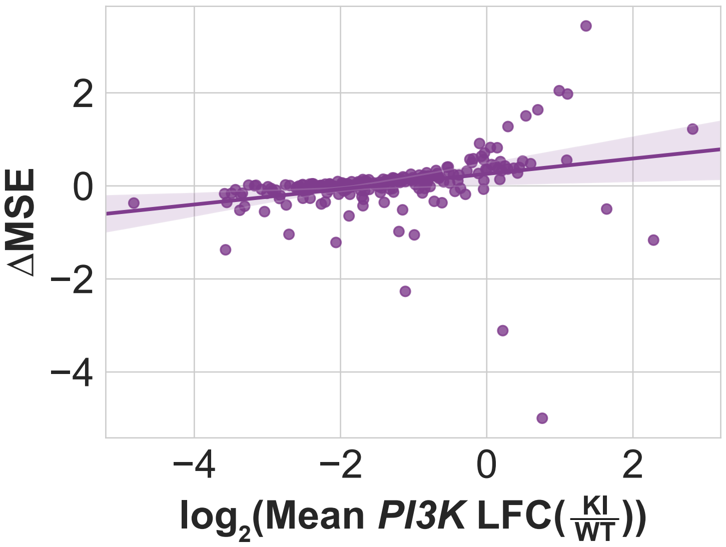

Fig. S2. Correlation between the mean LFC between the *PI3K* KI and WT conditions for each gene and the ΔMSE of its trained ensemble model from the null model. Spearman's ρ=0.636, p=5.4e-26

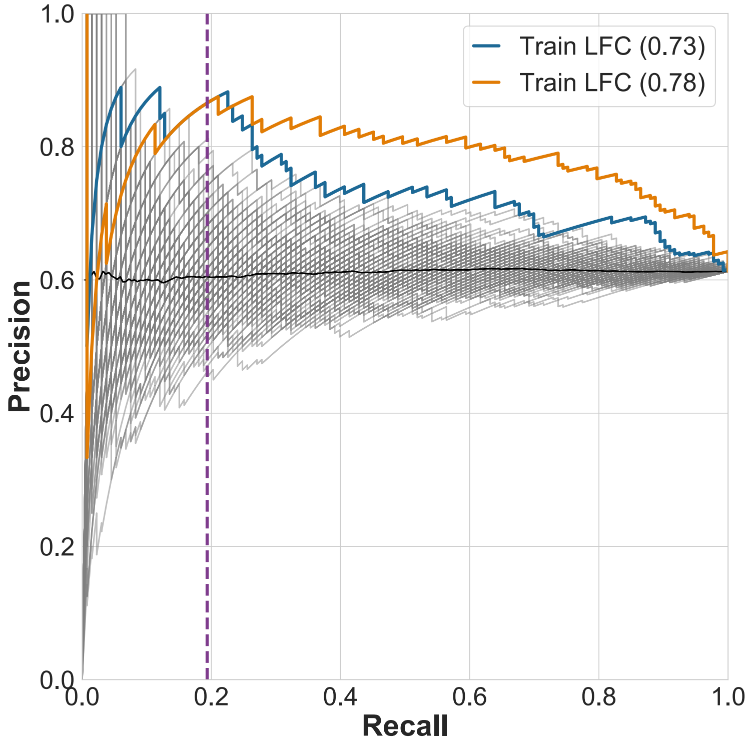

Fig. S3. Higher ranked models are more likely to be more predictive than the null model. AUPR curve plot of different methods for sorting gene predictions. Sorting by mean LFC between the training conditions places more accurate predictions at the top of the list. The threshold for selecting more accurate predictions (purple, dashed line) was calculated using the elbow rule of the sorted mean LFC values in the training condition.

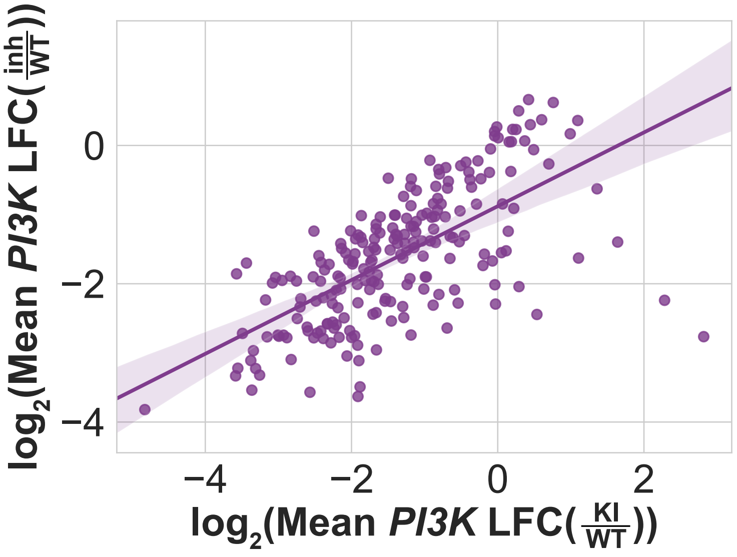

Fig. S4. Correlation between the mean LFC between the *PI3K* KI and WT conditions and the mean LFC between the $PI3K^{\text{inh}}$ and WT conditions for each gene. Spearman's ρ=0.684, p=2.7e-31

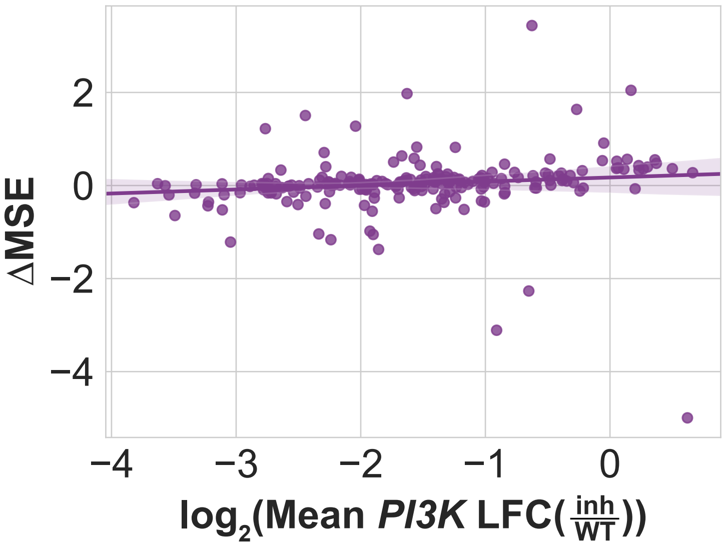

Fig. S5. Correlation between the mean LFC between the *PI3K*^{inh} and WT conditions for each gene and the ΔMSE of its trained ensemble model from the null model. Spearman's ρ=0.418, p=1.4e-10

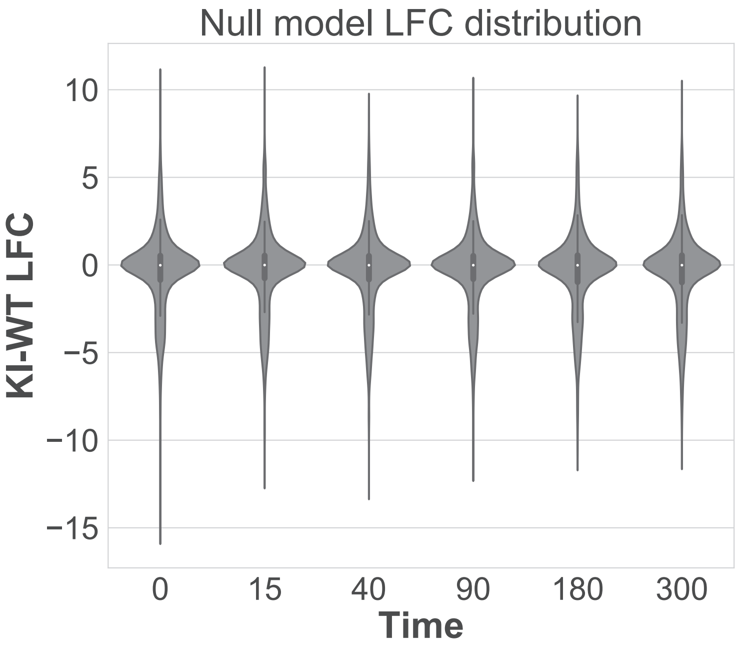

Fig. S6. Distributions of predicted LFC values between KI and WT conditions at each time by the simulation library. On average, the library predicts near zero LFC values at each time. However, there is large variation within the library, so the set of models DiffExPy matches to each gene outperform a randomly selected model.

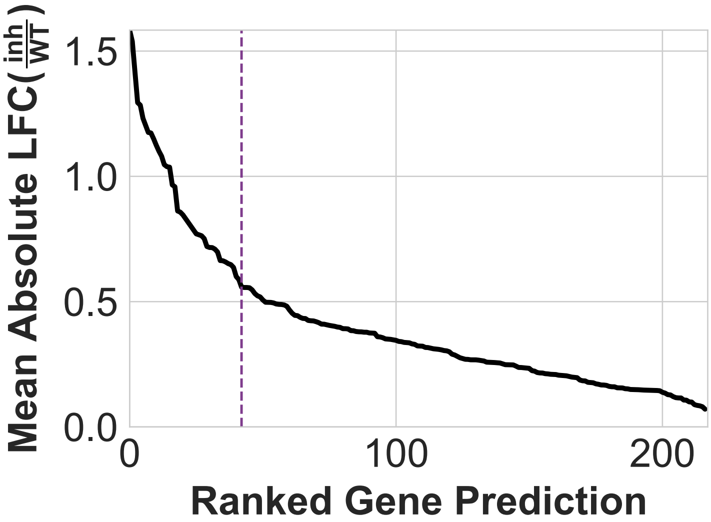

Fig. S7. The 217 trained gene models were ranked by the mean absolute LFC of each time point between the $PI3K^{\text{inh}}$ and WT conditions. The purple, dashed line shows the cutoff for the top set of genes using the elbow rule.

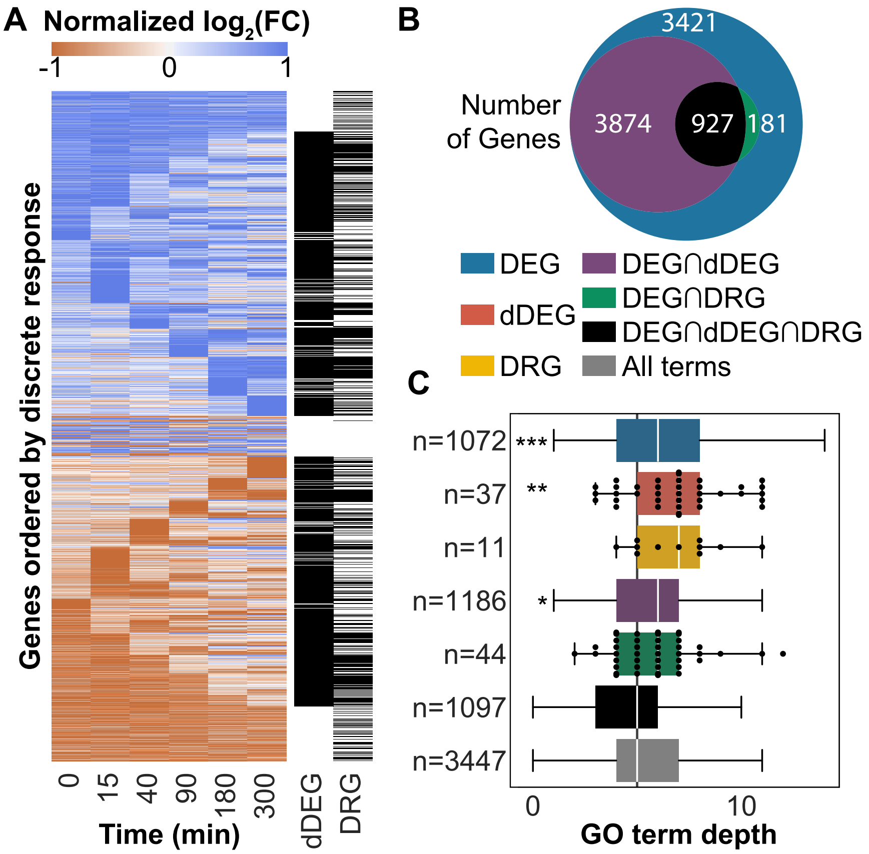

Fig. S8. Summary of gene classifications comparing *PI3K* KI (H1047R) to WT. (A) Heatmap of row normalized LFC for all DEGs. Genes that were classified as dDEG or DRG are also labeled. (B) Overlap of genes that were classified as DEG, dDEG, DRG, or some combination. By definition, all dDEGs and DRGs must also be DEGs. (C) Comparison of distributions of GO term depths uniquely associated with intersections of gene sets. p-values were calculated using a discrete KS test. * $p< 0.05$, ** $p< 0.01$, *** $p< 0.001$.

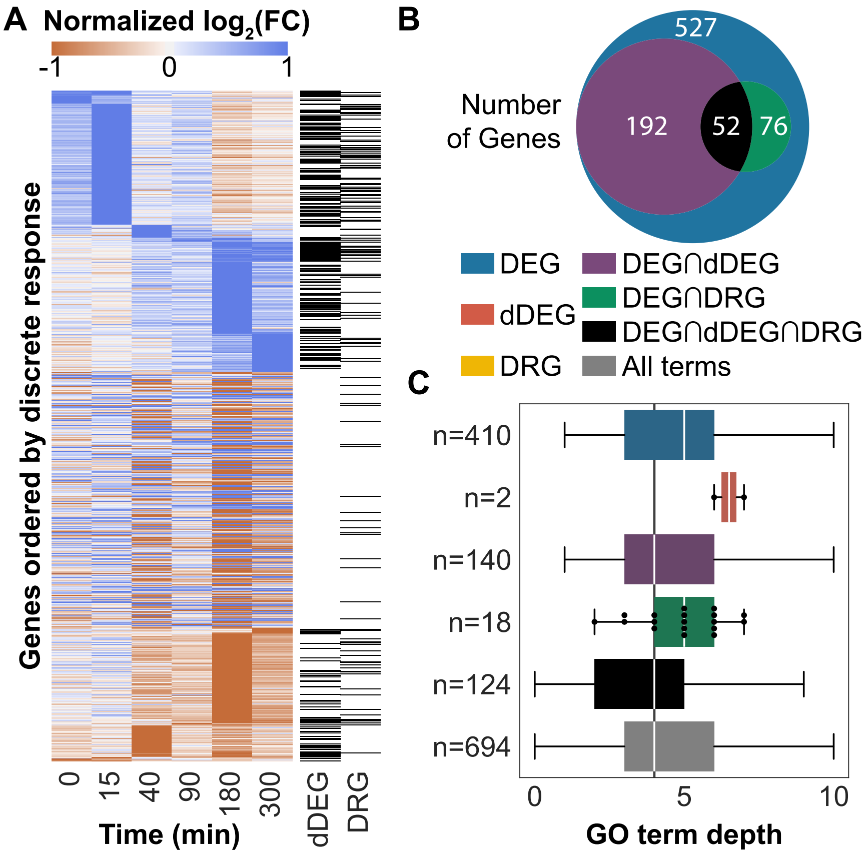

Fig. S9. Summary of gene classifications comparing $PI3K^{\mathrm{inh}}$ (A66 treatment) to WT. Few genes were significantly impacted by the $PI3K^{\mathrm{inh}}$, which limits how many GO terms can be found. (A) Heatmap of row normalized LFC for all DEGs. Genes that were classified as dDEG or DRG are also labeled. (B) Overlap of genes that were classified as DEG, dDEG, DRG, or some combination. By definition, all dDEGs and DRGs must also be DEGs. (C) Comparison of distributions of GO term depths uniquely associated with intersections of gene sets.

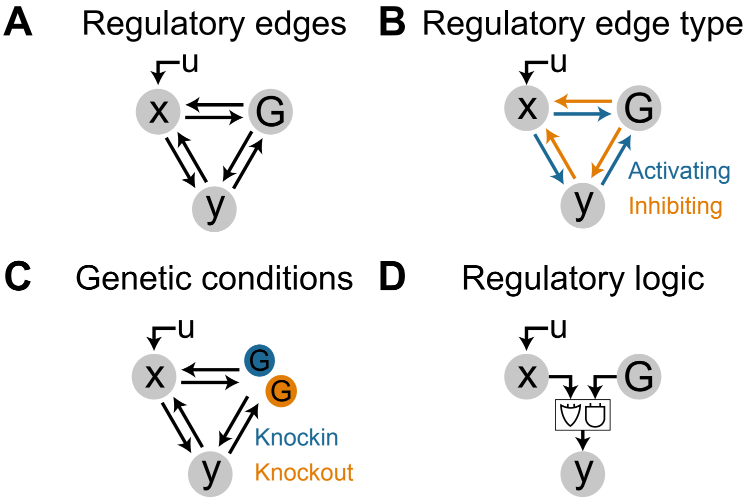

Fig. S10. In silico simulation library model constraints. (A) All unique combinations of three-node networks without self-edges were generated. Node y is the output node whose simulated expression is matched to discrete trajectories of genes in the experimental data. Node G is the experimentally perturbed node. Node x represents interactions with the rest of the genome. A forcing function, u, is applied to node x to simulate the application of a treatment. (B) Edges between nodes can be activating or inhibiting. (C) Simulations were conducted where node G is knocked out (no expression in the simulation) or knocked in (constituitively expressed in the simulation). (D) If there are two upstream regulators of a node, combinations of the regulators interacting with AND or OR logic were created.

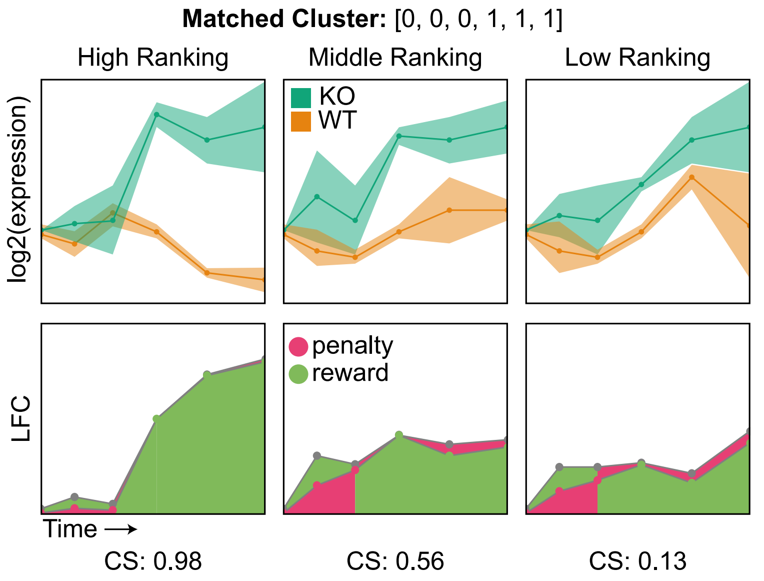

Fig. S11. Cluster score of genes or model simulations. The high ranking gene (left) has a cluster score (CS) close to 1 because it has a low LFC at the early time points and highly significant LFC at later time points which match the assigned cluster. The low ranking gene (right) still qualitatively matches the assigne cluster. However, it has a lower CS because the LFC values at early time points are higher and the LFC values at later time points are not as significant.

References

[1] Vladimir Yu Kiselev, Veronique Juvin, Mouhannad Malek, Nicholas Luscombe, Phillip Hawkins, Nicolas Le Novère, and Len Stephens. Perturbations of pip3 signalling trigger a global remodelling of mrna landscape and reveal a transcriptional feedback loop. *Nucleic acids research*, 43(20):9663–9679, 2015.

[2] Mark D Robinson, Davis J McCarthy, and Gordon K Smyth. edger: a bioconductor package for differential expression analysis of digital gene expression data. *Bioinformatics*, 26(1):139–140, 2010.

[3] Matthew E Ritchie, Belinda Phipson, Di Wu, Yifang Hu, Charity W Law, Wei Shi, and Gordon K Smyth. limma powers differential expression analyses for rna-sequencing and microarray studies. *Nucleic acids research*, 43(7):e47, 2015.

[4] Charity W Law, Yunshun Chen, Wei Shi, and Gordon K Smyth. voom: Precision weights unlock linear model analysis tools for rna-seq read counts. *Genome biology*, 15(2):R29, 2014.

[5] Thomas Schaffter, Daniel Marbach, and Dario Floreano. Genenetweaver: in silico benchmark generation and performance profiling of network inference methods. *Bioinformatics*, 27(16):2263–2270, 2011.

[6] Michael Ashburner, Catherine A Ball, Judith A Blake, David Botstein, Heather Butler, J Michael Cherry, Allan P Davis, Kara Dolinski, Selina S Dwight, Janan T Eppig, et al. Gene ontology: tool for the unification of biology. *Nature genetics*, 25(1):25, 2000.

[7] Taylor B Arnold and John W Emerson. Nonparametric goodness-of-fit tests for discrete null distributions. *R Journal*, 3(2):34–39, 2011.

[8] Marcel H Schulz, William E Devanny, Anthony Gitter, Shan Zhong, Jason Ernst, and Ziv Bar-Joseph. Drem 2.0: Improved reconstruction of dynamic regulatory networks from time-series expression data. *BMC systems biology*, 6(1):104, 2012.

[9] Ronald A Fisher. On the interpretation of χ 2 from contingency tables, and the calculation of p. *Journal of the Royal Statistical Society*, 85(1):87–94, 1922.

[10] Stéfan van der Walt, S Chris Colbert, and Gael Varoquaux. The numpy array: A structure for efficient numerical computation. *Computing in Science Engineering*, 13(2):22–30, March 2011.

[11] Wes McKinney. pandas: a foundational python library for data analysis and statistics. *Python for High Performance and Scientific Computing*, pages 1–9, 2011.

[12] DV Klopfenstein, Liangsheng Zhang, Brent S Pedersen, Fidel Ramírez, Alex Warwick Vesztrocy, Aurélien Naldi, Christopher J Mungall, Jeffrey M Yunes, Olga Botvinnik, Mark Weigel, et al. Goatools: A python library for gene ontology analyses. *Scientific reports*, 8(1):10872, 2018.

[13] J. D. Hunter. Matplotlib: A 2d graphics environment. *Computing in Science Engineering*, 9(3):90–95, May 2007.
